# Phase 2 study of regorafenib in patients with progressive glioblastoma after failure of bevacizumab

**DOI:** 10.1093/noajnl/vdag144

**Published:** 2026-06-09

**Authors:** Alyssa Lucas, David Peereboom, Yasmeen Rauf, Hong Li, Joshua Hanlon, Manmeet Ahluwalia

**Affiliations:** Rose Ella Burkhardt Brain Tumor & Neuro-Oncology Center, Cleveland Clinic Foundation, Cleveland, OH, USA; Lerner Research Institute Quantitative Health Sciences, Cleveland Clinic Foundation, Cleveland, OH, USA; Rose Ella Burkhardt Brain Tumor & Neuro-Oncology Center, Cleveland Clinic Foundation, Cleveland, OH, USA; Lerner Research Institute Quantitative Health Sciences, Cleveland Clinic Foundation, Cleveland, OH, USA; Rose Ella Burkhardt Brain Tumor & Neuro-Oncology Center, Cleveland Clinic Foundation, Cleveland, OH, USA; Rose Ella Burkhardt Brain Tumor & Neuro-Oncology Center, Cleveland Clinic Foundation, Cleveland, OH, USA

**Keywords:** anti-angiogenesis, bevacizumab-refractory glioblastoma, glioblastoma, regorafenib, targeted therapy

## Abstract

**Background:**

Treatment options in progressive glioblastoma (GBM) are limited, especially in patients who have experienced disease progression while receiving bevacizumab therapy. This phase 2 study was conducted to evaluate the efficacy and safety of regorafenib monotherapy in patients with GBM.

**Methods:**

Eligible patients had radiographic progression of histologically confirmed GBM following second- or third-line bevacizumab therapy. They received regorafenib monotherapy using the dose escalation strategy demonstrated in the ReDOS phase 2 clinical trial evaluating regorafenib in metastatic colon cancer. The target dose was 160 mg once daily, administered for 3 weeks on/1 week off in 28-day cycles. Patients underwent brain magnetic resonance imaging (MRI) every 8 weeks and were assessed via modified Response Assessment in Neuro-Oncology criteria. Patients continued treatment with regorafenib therapy until they experienced progression or excessive toxicity.

**Results:**

Based on the analysis of 13 enrolled patients, there were no responses and 12 of 13 (92.3%) had progressive disease on brain MRI; 1 patient went off study due to an adverse event occurring before assessment. The median overall survival (OS) was 4.1 months (95% CI 2.0-7.3). The OS at 12 months was 23% (0.2-46). The PFS at both 3 and 6 months was 7.7% (0.0-22.2%) with a median PFS of 1.0 month, (0.4-1.8). There was a total of 14 Grade 3 or 4 AEs and no Grade 5 AEs.

**Conclusions:**

Regorafenib did not improve OS or PFS in bevacizumab-refractory recurrent glioblastoma. The study was halted early due to futility.

**Trial Registration:**

NCT04051606.

Key Points

- This study investigated the use of regorafenib monotherapy in bevacizumab-refractory progressive glioblastoma
- The study was halted early due to futility
- Regorafenib was ineffective in GBM patients after the failure of bevacizumab

Importance of the Study

Treatment options for progressive glioblastoma (GBM) are more limited than for up-front treatment, especially for patients who have experienced progression while receiving bevacizumab therapy where no study has yet demonstrated meaningful efficacy. In this phase 2 single-center study, patients with glioblastoma refractory to bevacizumab therapy received the oral multityrosine kinase inhibitor regorafenib. Unfortunately, there was no improvement in progression-free survival or overall survival with the addition of regorafenib therapy. This is the first prospective clinical trial to evaluate the efficacy of regorafenib in progressive, bevacizumab-refractory GBM. Unfortunately, this study was halted early due to futility. This study does not support further exploration of the use of regorafenib in this setting.

Glioblastoma (GBM) is the most common primary malignant brain tumor in adults.^1^ The standard of care for initial GBM treatment has consisted of maximal safe surgical resection followed by concurrent chemoradiation with temozolomide followed by adjuvant temozolomide chemotherapy and possibly with tumor treating fields.^2^^,^^3^ However, virtually all patients experience progressive disease following initial treatment and the median overall survival (OS) remains less than 2 years.^2^ In the second- and third-line treatment setting treatment options for disease progression are more limited, and the guidelines are less clear.

GBMs are highly vascularized tumors and like many other solid tumors, angiogenesis is crucial for their survival and growth. Bevacizumab is a monoclonal antibody targeting the angiogenic cytokine vascular endothelial growth factor (VEGF) that has proven effective when combined with chemotherapy in the treatment of colorectal, non–small cell lung, and breast cancers—leading to FDA approval for those indications.^4-6^ It is also often used as a second-line treatment for GBM per the National Comprehensive Cancer Network (NCCN) guidelines and has been FDA-approved for use in progressive GBM, although thus far has demonstrated an improvement in progression-free survival (PFS) but not in OS, either as monotherapy or in combination with other chemotherapy agents such as irinotecan.^7-9^ In addition, third-line agents to be used after progression with bevacizumab treatment (so-called bevacizumab-refractory recurrences) are even more sparse.

Regorafenib is an oral small molecule inhibitor of multiple tyrosine kinases that are implicated in tumorigenesis, including angiogenesis with VEGF inhibition.^10^ Regorafenib has demonstrated efficacy in overall and progression-free survival in other solid tumors, including hepatocellular carcinoma, colorectal cancer, and gastrointestinal stromal tumor (GIST).^11-13^ Promising preclinical data led to the phase 2 prospective randomized REGOMA trial, which evaluated regorafenib in the second-line GBM treatment setting (at the time of first progression), notably prior to receiving bevacizumab therapy.^10^^,^^14^^,^^15^ Patients in this trial were randomized to receive either regorafenib or lomustine, a commonly used nitrosourea in recurrent GBM. The median OS in the regorafenib group was statistically significantly longer than that in the lomustine group (7.4 months in regorafenib, 5.6 months lomustine; *P* = .0009). Given these data, regorafenib was added to the NCCN guidelines for second-line GBM treatment, along with bevacizumab, nitrosourea therapy, and tumor treating fields.^16^ The REGOMA-OSS observational study yielded similar survival and safety results, reporting an OS of 7.9 months in patients receiving regorafenib as second-line treatment—patients were also excluded from this study if they had previously received bevacizumab.^17^

A retrospective study from Turkey evaluated the efficacy of regorafenib in the third line setting for patients who have progressed despite bevacizumab therapy. Median OS was shorter in this setting, reported as 4.1 months with median PFS 2.5 months.^18^ No therapies have demonstrated a survival benefit in third-line, bevacizumab-refractory GBM; this study was designed to evaluate the efficacy of regorafenib in this setting.

## Methods

### Patient Population

Eligible patients were ≥18 years of age with histologically confirmed glioblastoma (according to the WHO 2016 classification of tumors of the central nervous system) with Karnofsky Performance Score (KPS) of ≥70 and adequate bone marrow, renal, and liver function. Patients were required to have experienced progression following initial surgical resection and subsequent radiation and temozolomide chemotherapy, in addition to having documented radiographic progression following bevacizumab therapy for progressive glioblastoma. Patients were required to have received bevacizumab or a bevacizumab-containing regimen as the immediate prior treatment regimen before study enrollment. Patients were eligible if they had experienced up to three prior treatment failures and could have received bevacizumab at either first or second progression. Primary exclusion criteria included prior regorafenib treatment, clinically significant cardiac disease, uncontrolled concomitant medical conditions, and major surgery in the 4 weeks prior to starting study treatment. Other concurrent cancer-directed therapy was not allowed while on study. For full eligibility criteria, please see Supplementary Material S1 (trial registration number NCT04051606) in the online supplementary material. All patients provided written informed consent after being offered study entry by the treating physician. The protocol was approved by the Institutional Review Board (IRB) at the Case Comprehensive Cancer Center.

### Study Design

This phase 2 study was designed to evaluate the median OS and PFS in patients with recurrent or progressive GBM who have progressed on bevacizumab or after completion of a bevacizumab-containing regimen if there was no intervening systemic therapy between completion of bevacizumab and study enrollment. Participants received oral regorafenib using the dose escalation strategy in the ReDOS phase 2 clinical trial of regorafenib in metastatic colon cancer.^19^ The target dose was 160 mg once daily and regorafenib was administered for 3 weeks on/1 week off in 28-day cycles. Predetermined dose reductions to 120 mg and 80 mg were utilized based on grade of toxicity, and if more than two dose reductions were required, regorafenib would be discontinued altogether. Specific criteria for dose adjustments and discontinuation are described in the protocol (Supplementary Material S2 in the online supplementary material). Patients underwent baseline gadolinium contrast-enhanced MRI prior to starting regorafenib and then repeated brain MRI every 8 weeks. Imaging was evaluated based on the modified Response Assessment in Neuro-Oncology (RANO), including use of fluid-attenuated inversion recovery sequences and clinical status given the possibility of angiogenesis inhibition altering the appearance of contrast-enhanced images.^20^ Blood studies were obtained to ­monitor for toxicity, including complete blood count with differential and comprehensive metabolic panel at least once at the beginning of each cycle. All adverse events (AEs) described by Common Terminology Criteria for Adverse Events (CTCAE) version 5.0 were recorded.^21^ Participants continued regorafenib until they experienced either progression or toxicity.

### Study Endpoints

The primary endpoint was median OS in GBM patients with disease progression on bevacizumab or after completion of a bevacizumab-containing regimen if there was no intervening systemic therapy between completion of bevacizumab and study enrollment. OS was defined as time from treatment initiation until death of any cause. Secondary endpoints included safety and tolerability of regorafenib described using CTCAE v5.0, objective response rate by modified RANO criteria, and progression-free survival at 6 months (PFS-6).

### Statistical Design and Analysis

A one stage accrual design with an accrual goal of 22 evaluable patients was employed to test the hypothesis that regorafenib can increase the overall median survival from 4 months to 7 months. The baseline survival of 4 months was derived from datasets in GBM patients who have experienced failure of bevacizumab.^22^^,^^23^ The goal of 7 months survival was felt to be a meaningful improvement in outcomes for this patient group and approximates the OS achieved in the REGOMA trial.^15^ Assuming overall survival follows an exponential distribution, accrual takes approximately 12 months, and there are 12 months of additional follow-up once accrual has been completed, there will be 81% power to detect the specified difference using a two-sided test with 10% type I error. Patient characteristics at treatment initiation and reasons for discontinuing treatment were summarized descriptively using median (range) and frequency (percentage). PFS (progression or death) and OS were analyzed via the Kaplan-Meier method. Treatment-related AEs were reported by organ system and preferred term in the highest grade at patient level.

## Results

### Patient Characteristics

A total of 14 patients registered for this study; 13 patients were included in the analysis (one patient excluded prior to treatment initiation due to ineligibility). Patient characteristics are summarized in Table 1. The median age was 66 years (range 36-70) and median disease duration was 1.5 years (range 0.96-6.1). All patients had received concurrent radiation therapy and temozolomide, and 12/13 had received adjuvant temozolomide. The median number of treatment failures prior to the study was 2 (range 1-3). At the time of enrollment, four patients had a KPS of 90, six with KPS 80, and three with KPS 70. All patients had IDH (isocitrate dehydrogenase) wildtype tumors. MGMT methylation status was not hypermethylated: 9; hypermethylated: 4, and unknown: 1. Complete patient baseline characteristic data can be found in Table 1. In addition to study-mandated radiation, temozolomide, and bevacizumab, prior therapies included: lomustine (five patients), etoposide (two), and one patient each: capecitabine, nivolumab, tumor treating fields, and stereotactic radiosurgery.

**Table 1.** Baseline patient characteristics

Variable	Value; total *n* = 13
Age in years, median (range)	66 (36-70)
Female sex, *n* (%)	6 (46)
Disease duration in years, median (range)	1.5 (0.96-6.1)
KPS, *n* (%)	
70	3 (23)
80	6 (46)
90	4 (31)
MGMT methylation status, *n* (%)	
Hypermethylated	4 (31)
Not hypermethylated	9 (69)

### Efficacy

**Figure 1.**
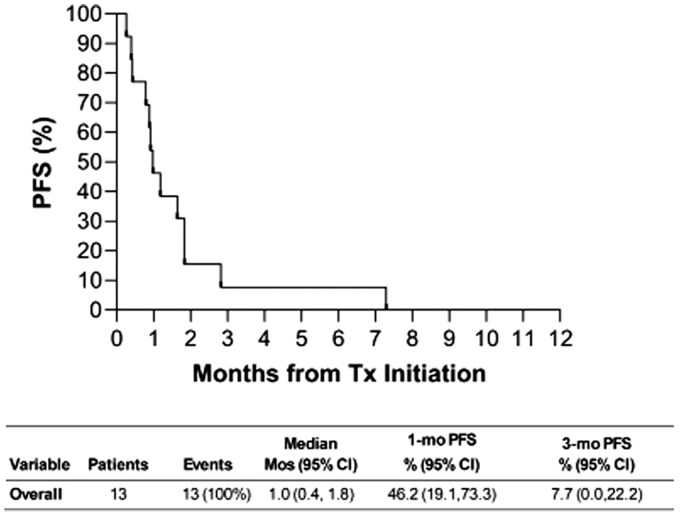
Progression-free survival (Kaplan-Meier curve).

**Figure 2.**
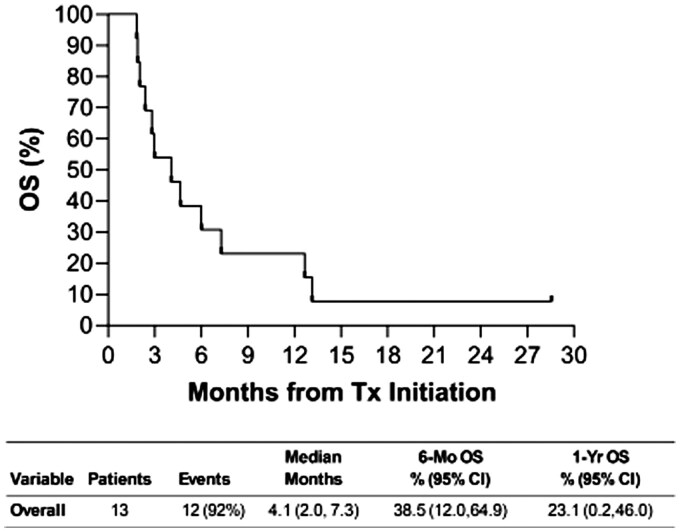
Overall survival (Kaplan-Meier curve).

The median follow-up time was 4.1 (interquartile range 2.4, 7.3) months with an overall range 1.8-28 months. Based on the analysis of 13 patients, there were no responses and 12 of 13 (92%) had progressive disease (PD) on brain MRI; 1 patient went off treatment due to an adverse event (AE) occurring before assessment. The overall median PFS was 1.0 months; 54% of patients experienced rapid deterioration that prompted early MRIs—at 1 month instead of the planned 2-month interval—that confirmed progressive disease. PFS was 46% (95% CI, 19%-73%) at 1 month and 7.7% (0.0%-22%) at both 3 and 6 months, as depicted in Figure 1. Median OS was 4.1 months (95% CI 2.0-7.3); 6-month OS was 38% (95% CI 12-65), depicted in Figure 2. One participant had PD at 1.8 months and was taken off study but remained alive at last follow-up at 28 months from treatment initiation. There were no differences in outcomes according to MGMT promoter methylation status although the study was underpowered to detect such a difference. Due to rapid progression seen in the study patients, a decision was made to halt the trial early.

### Safety and Toxicity

All reported treatment-related AEs are listed in Table S3, and Grade 3-4 and 5 AEs in Table 2. There was a total of 14 Grade 3 or 4 AEs per CTCAE 5.0 criteria, including 4 related to weakness (2 focal, 2 generalized), 3 with fatigue, 3 with hepatic dysfunction (including 1 with liver failure), and 1 patient each with pancreatitis, thrombocytopenia, palmar-plantar erythrodysesthesia syndrome, and thromboembolic event. There were no grade 5 treatment-related AEs. The most common adverse effect of any grade was fatigue. None of the participants required dose reductions or interruptions due to toxicity.

**Table 2.** Grade 3-4 and grade 5 treatment-related adverse effects

Adverse Effect *N* = 13 patients	Grade 3-4 *N* = patients	Grade 5	Relationship to treatment
Pancreatitis	1	0	Unlikely
Fatigue	3	0	Possible
Hepatic failure	1	0	Possible
Alanine aminotransferase increased	1	0	Probable
Aspartate aminotransferase increased	1	0	Probable
Platelet count decreased	1	0	Possible
Generalized weakness	2	0	Possible
Right-sided weakness	2	0	Unlikely
Palmar-plantar erythrodysesthesia syndrome	1	0	Possible
Thromboembolic event	1	0	Unlikely

## Discussion

To our knowledge, this is the first prospective clinical trial to evaluate the efficacy of regorafenib in progressive, bevacizumab-refractory GBM. No new safety signals emerged during the study, but regorafenib did not demonstrate an improvement in PFS or OS. Due to rapid progression seen in the study patients, a decision was made to halt the trial early.

The REGOMA and REGOMA-OSS trials also evaluated the efficacy of regorafenib in progressive glioblastoma, however patients who had already received or progressed on bevacizumab therapy were excluded from these studies.^15^^,^^17^ This exclusion likely accounts for the lower OS and PFS in this article comparatively. The results of this article are more comparable to the retrospective study of regorafenib use in patients with bevacizumab-refractory GBM, which reported median PFS 2.5 months and OS 4.1 months.^19^ The retrospective study by Tünbekici described patients with GBM who had received prior bevacizumab.^18^ That study suggested some activity in the setting of bevacizumab failure. The median age of the population in that cohort, however, was 53 years while in this study the median age was 66 years, suggesting that younger patients might have better responsiveness to regorafenib.

This study has limitations. The study was small, underpowered, and did not reach full accrual. The early termination was based on the consensus from the treating team that accrual of additional patients would not be ethical. In addition, it was conducted at a single center, perhaps limiting the generalizability of the results.

Several reasons could explain the lack of efficacy of regorafenib in this study. First, by virtue of the study eligibility, patients had experienced bevacizumab failure and thus entered this study with GBMs refractory to VEGF inhibition. The lack of activity of regorafenib suggests that, in the context of glioblastoma, regorafenib also acts primarily through VEGF inhibition and not through inhibition of other kinases that serve as targets of this agent. Second, the failure of regorafenib in this study and indeed essentially all other efforts to treat glioblastoma after bevacizumab failure, is the often rapid “rebound” edema, contrast-enhancement, and neurologic decline seen after withdrawal of bevacizumab.^24^ Although bevacizumab and regorafenib both interfere with VEGF signaling, regorafenib appears in this study to be inadequate to control this rebound phenomenon. Third, the dose escalation strategy in this trial, as was used in the ReDOS study, may have caused a substantial delay in reaching the optimal dose of regorafenib.^19^ Unfortunately, effective treatment of glioblastoma in the setting of bevacizumab failure remains elusive.

Regorafenib, bevacizumab, temozolomide, lomustine, combination procarbazine/lomustine/vincristine regimens, and tumor treating fields are all preferred options for progressive GBM according to the NCCN guidelines; however, current data reflect benefit from these regimens at the first progression.^16^ Although regorafenib has activity in bevacizumab-naive patients and this study has several limitations, this trial should give pause to clinicians contemplating use of this agent in patients for whom bevacizumab has failed. There remains a lack of and need for prospective data for effective treatments beyond the first progression or after bevacizumab therapy.

## Supplementary Material

vdag144_Supplementary_Data

## Data Availability

All data analyzed from this study will be made available upon reasonable request.
