# Supplementary material for: Phase 2 study of regorafenib in patients with progressive glioblastoma after failure of bevacizumab
Source: Neurooncol Adv. 2026 Jun 9;8(1):vdag144. doi: 10.1093/noajnl/vdag144 (PMC13275312; doi:10.1093/noajnl/vdag144)
Supplement: vdag144_Supplementary_Data [file vdag144_supplementary_data.docx]

**Phase 2 study of regorafenib in patients with progressive glioblastoma after failure of bevacizumab**

**Supplementary Information**

**Supplemental 1:** Full Eligibility Criteria

**Inclusion Criteria**

1. The participant (or legal representative if applicable) provides written informed consent for the trial.
2. Patients with histologically confirmed glioblastoma or other grade IV malignant glioma (i.e. gliosarcoma, small cell glioblastoma, etc.), recurrent after prior external-beam fractionated radiotherapy and temozolomide chemotherapy.
3. Patients with documented radiographic progression following bevacizumab therapy for treatment of glioblastoma
4. Patients with up to 3 prior recurrences are allowed (patients could have received bevacizumab or bevacizumab containing regimen either in first or second recurrence).
5. Karnofsky performance status ≥ 70%.
6. Age ≥ 18 years old.
7. Patients must have the following laboratory values: Absolute neutrophil count (ANC) ≥ 1.5 x 109/L, Platelets ≥ 100 x 109/L, Hemoglobin (Hgb) > 9 g/dL, Serum total bilirubin: ≤ 1.5 x ULN, ALT and AST ≤ 3.0 x ULN, Serum creatinine ≤ 1.5 x ULN, Blood coagulation parameters: INR ≤ 1.5
8. Minimum interval since completion of radiation treatment is 12 weeks
9. Minimum interval since last drug therapy: 3 weeks since last non-cytotoxic therapy; 3 weeks must have elapsed since the completion of a non-nitrosourea containing chemotherapy regimen or 6 weeks since the completion of a nitrosourea containing chemotherapy regimen.
10. Women of childbearing potential must have a negative serum pregnancy test performed within 7 days prior to the start of study drug. Post-menopausal women (defined as no menses for at least 1 year) and surgically sterilized women are not required to undergo a pregnancy test. The definition of adequate contraception will be based on the judgment of the investigator.
11. Subjects (men and women) of childbearing potential must agree to use adequate contraception beginning at the signing of the ICF until at least2 months after the last dose of study drug. The definition of adequate contraception will be based on the judgment of the principal investigator or a designated associate.
12. Patients must have no concurrent malignancy except curatively treated basal or squamous cell carcinoma of the skin or carcinoma in situ of the cervix and breast, adequately treated stage I or II cancer from which the patient is in complete remission. Patients with other prior malignancies must be disease-free for ≥ three years.
13. Patients must be maintained on a stable or decreasing corticosteroid regimen from the time of their baseline scan until the start of treatment and/or for at least 5 days before starting treatment. The maximum dosing of corticosteroid therapy is 4mg/day.
14. Life expectancy of at least 12 weeks (3 months).
15. Subject must be able to swallow and retain oral medication.

**Exclusion Criteria**

1. Patients who have had previous treatment with Regorafenib
2. Patients who have undergone major surgery (e.g. intra-thoracic, intra-abdominal or intra-pelvic), open biopsy or significant traumatic injury ≤ 4 weeks prior to starting study drug, or patients who have had minor procedures, percutaneous biopsies or placement of vascular access device ≤ 1 week prior to starting study drug, or who have not recovered from side effects of such procedure or injury
3. Patients with impaired cardiac function or clinically significant cardiac diseases, including any of the following:
   1. Congestive heart failure – New York Heart Association (NYHA) > Class II
   2. History or presence of serious uncontrolled ventricular arrhythmias. Cardiac arrhythmias requiring anti-arrhythmic therapy other than beta blockers or digoxin.
   3. Clinically significant resting bradycardia (defined as bradycardia that required intervention)
   4. Active coronary artery disease defined as Any of the following within 6 months prior to starting study drug: myocardial infarction (MI), severe/unstable angina, Coronary Artery Bypass Graft (CABG)
   5. Cerebrovascular Accident (CVA), Transient Ischemic Attack (TIA), Pulmonary Embolism (PE) in the last 6 months
   6. Uncontrolled hypertension (defined by a SBP ≥ 160 mm Hg or DBP ≥ 100 mm Hg despite anti-hypertensive medications)
4. Patients with cirrhosis, or active viral or nonviral hepatitis.
5. Known diagnosis of human immunodeficiency virus (HIV) infection (HIV testing is not mandatory)
6. Other concurrent severe and/or uncontrolled concomitant medical conditions (e.g. active or uncontrolled infection, uncontrolled diabetes) that could cause unacceptable safety risks or compromise compliance with the protocol
7. Pregnant or breast-feeding women
8. Patients with known hypersensitivity to Chinese hamster ovary cell products or other recombinant human, chimeric, or humanized antibodies
9. Patients with active bleeding or pathologic conditions that carry a high risk of bleeding, (i.e. hereditary hemorrhagic telangiectasia).
10. Patients who are currently receiving anticoagulation treatment (warfarin is not allowed, low weight heparin is allowed). Evidence or history of bleeding diathesis or coagulopathy.
11. Patients unwilling or unable to comply with the protocol
12. Any hemorrhage or bleeding event ≥ NCI CTCAE v5.0 Grade 3 within 4 weeks prior to start of study medication.
13. Patients with phaeochromocytoma.
14. Ongoing infection > Grade 2 NCI-CTCAE v5.0.
15. Presence of a non-healing wound, non-healing ulcer, or bone fracture.
16. Persistent proteinuria: Grade 3 NCI-CTCAE v5.0 (> 3.5 g/24 hrs, measured by urine protein: creatinine ratio on a random urine sample).
17. Interstitial lung disease with ongoing signs and symptoms at the time of informed consent.
18. Pleural effusion or ascites that causes respiratory compromise (≥ NCI-CTCAE version 5.0 Grade 2 dyspnea).
19. History of organ allograft (including corneal transplant).
20. Known or suspected allergy or hypersensitivity to any of the study drugs, study drug classes, or excipients of the formulations given during the course of this trial.
21. Any malabsorption condition.
22. Women who are pregnant or breast-feeding.
23. Any condition which, in the investigator’s opinion, makes the subject unsuitable for trial participation.

**Supplemental 2:** Dose adjustments per study protocol

The starting dose of regorafenib is 80 mg once daily. Weekly dose escalation if no significant drug-related toxicities, up to 160 mg/day. The dose escalation may be performed at a greater interval than one week at the discretion of the investigator based on the tolerability of the drug. Study medication will be administered on a 3 weeks on/1week off schedule [3 weeks out of every 4].

Doses will be delayed or reduced for clinically significant hematologic and non-hematologic toxicities that are related to protocol therapy according to the guidelines shown in the Dose Delays/Dose Modifications table that follows. Dose modifications will follow predefined dose levels. Dose adjustments for hematologic toxicity are based on the blood counts obtained in preparation for the day of treatment.

If a dose reduction has been performed, intra-subject dose re-escalation can be considered (up to the maximal 160 mg daily dose) at the discretion of the treating physician provided that the toxicity (ies) has resolved to baseline.

The modifications of regorafenib will follow the following predefined dose levels:

| Dose level 0 (standard starting dose) | 160 mg once daily | Four 40 mg tablets of regorafenib |
| --- | --- | --- |
| Dose level -1 | 120 mg once daily | Three 40 mg tablets of regorafenib |
| Dose level -2 | 80 mg once daily | Two 40 mg tablets of regorafenib |

**Supplemental 3:** Treatment-Related Adverse Effects (AEs). Number of reported adverse effects, reported as n (%).

| **System Organ Class/**  **Adverse Effect** | **Overall** | **Gr1-2** | **Gr3-4** | **Gr5** |
| --- | --- | --- | --- | --- |
| **Gastrointestinal disorders** | **5 (38.5%)** | **4 (30.8%)** | **1 (7.7%)** | **0 (0.0%)** |
| Diarrhea | 2 (15.4%) | 2 (15.4%) | 0 (0.0%) | 0 (0.0%) |
| Mucositis oral | 1 (7.7%) | 1 (7.7%) | 0 (0.0%) | 0 (0.0%) |
| Nausea | 1 (7.7%) | 1 (7.7%) | 0 (0.0%) | 0 (0.0%) |
| Vomiting | 1 (7.7%) | 1 (7.7%) | 0 (0.0%) | 0 (0.0%) |
| gum pain | 1 (7.7%) | 1 (7.7%) | 0 (0.0%) | 0 (0.0%) |
| pancreatitis | 1 (7.7%) | 0 (0.0%) | 1 (7.7%) | 0 (0.0%) |
| **General disorders and administration site conditions** | **8 (61.5%)** | **5 (38.5%)** | **4 (30.8%)** | **0 (0.0%)** |
| Edema face | 1 (7.7%) | 1 (7.7%) | 0 (0.0%) | 0 (0.0%) |
| Fatigue | 6 (46.2%) | 3 (23.1%) | 3 (23.1%) | 0 (0.0%) |
| Pain | 1 (7.7%) | 1 (7.7%) | 0 (0.0%) | 0 (0.0%) |
| weakness | 1 (7.7%) | 0 (0.0%) | 1 (7.7%) | 0 (0.0%) |
| **Hepatobiliary disorders** | **1 (7.7%)** | **0 (0.0%)** | **1 (7.7%)** | **0 (0.0%)** |
| Hepatic failure | 1 (7.7%) | 0 (0.0%) | 1 (7.7%) | 0 (0.0%) |
| **Infections and infestations** | **1 (7.7%)** | **1 (7.7%)** | **0 (0.0%)** | **0 (0.0%)** |
| Thrush | 1 (7.7%) | 1 (7.7%) | 0 (0.0%) | 0 (0.0%) |
| **Investigations** | **2 (15.4%)** | **2 (15.4%)** | **1 (7.7%)** | **0 (0.0%)** |
| Alanine aminotransferase increased | 2 (15.4%) | 1 (7.7%) | 1 (7.7%) | 0 (0.0%) |
| Aspartate aminotransferase increased | 1 (7.7%) | 0 (0.0%) | 1 (7.7%) | 0 (0.0%) |
| Blood bilirubin increased | 1 (7.7%) | 1 (7.7%) | 0 (0.0%) | 0 (0.0%) |
| Platelet count decreased | 1 (7.7%) | 0 (0.0%) | 1 (7.7%) | 0 (0.0%) |
| **Metabolism and nutrition disorders** | **1 (7.7%)** | **1 (7.7%)** | **0 (0.0%)** | **0 (0.0%)** |
| Hyponatremia | 1 (7.7%) | 1 (7.7%) | 0 (0.0%) | 0 (0.0%) |
| **Musculoskeletal and connective tissue disorders** | **2 (15.4%)** | **1 (7.7%)** | **1 (7.7%)** | **0 (0.0%)** |
| Generalized muscle weakness | 1 (7.7%) | 0 (0.0%) | 1 (7.7%) | 0 (0.0%) |
| Pain in extremity | 1 (7.7%) | 1 (7.7%) | 0 (0.0%) | 0 (0.0%) |
| **Nervous system disorders** | **3 (23.1%)** | **1 (7.7%)** | **2 (15.4%)** | **0 (0.0%)** |
| Headache | 1 (7.7%) | 1 (7.7%) | 0 (0.0%) | 0 (0.0%) |
| Muscle weakness right-sided | 2 (15.4%) | 0 (0.0%) | 2 (15.4%) | 0 (0.0%) |
| **Renal and urinary disorders** | **1 (7.7%)** | **1 (7.7%)** | **0 (0.0%)** | **0 (0.0%)** |
| Urinary frequency | 1 (7.7%) | 1 (7.7%) | 0 (0.0%) | 0 (0.0%) |
| **Respiratory, thoracic and mediastinal disorders** | **2 (15.4%)** | **2 (15.4%)** | **0 (0.0%)** | **0 (0.0%)** |
| Hoarseness | 1 (7.7%) | 1 (7.7%) | 0 (0.0%) | 0 (0.0%) |
| Voice alteration | 1 (7.7%) | 1 (7.7%) | 0 (0.0%) | 0 (0.0%) |
| **Skin and subcutaneous tissue disorders** | **1 (7.7%)** | **1 (7.7%)** | **1 (7.7%)** | **0 (0.0%)** |
| Pain of skin | 1 (7.7%) | 1 (7.7%) | 0 (0.0%) | 0 (0.0%) |
| Palmar-plantar erythrodysesthesia syndrome | 1 (7.7%) | 0 (0.0%) | 1 (7.7%) | 0 (0.0%) |
| Pruritus | 1 (7.7%) | 1 (7.7%) | 0 (0.0%) | 0 (0.0%) |
| Skin disorders - Other | 1 (7.7%) | 1 (7.7%) | 0 (0.0%) | 0 (0.0%) |
| **Vascular disorders** | **2 (15.4%)** | **1 (7.7%)** | **1 (7.7%)** | **0 (0.0%)** |
| Hypertension | 1 (7.7%) | 1 (7.7%) | 0 (0.0%) | 0 (0.0%) |
| Thromboembolic event | 1 (7.7%) | 0 (0.0%) | 1 (7.7%) | 0 (0.0%) |
